# Integrative application of heavy metal–resistant bacteria, moringa extracts, and nano-silicon improves spinach yield and declines its contaminant contents on a heavy metal–contaminated soil

**DOI:** 10.3389/fpls.2022.1019014

**Published:** 2022-11-15

**Authors:** Abdelsatar M. A. E. Eltahawy, El-Sayed A. M. Awad, Ahmed H. Ibrahim, Abdel-Rahman M. A. Merwad, El-Sayed M. Desoky

**Affiliations:** ^1^ Soil Science Department, Faculty of Agriculture, Zagazig University, Zagazig, Egypt; ^2^ Botany Department, Faculty of Agriculture, Zagazig University, Zagazig, Egypt

**Keywords:** spinach, moringa leaf extract, identification, heavy metal, antioxidant, chlorophyll

## Abstract

Microorganism-related technologies are alternative and traditional methods of metal recovery or removal. We identified and described heavy metal–resistant bacteria isolated from polluted industrial soils collected from various sites at a depth of 0–200 mm. A total of 135 isolates were screened from polluted industrial soil. The three most abundant isolate strains resistant to heavy metals were selected: *Paenibacillus jamilae* DSM 13815T DSM (LA22), *Bacillus subtilis* ssp. *spizizenii* DSM 15029T DSM (MA3), and *Pseudomonas aeruginosa* A07_08_Pudu FLR (SN36). A test was conducted to evaluate the effect of (1) isolated heavy metal–resistant bacteria (soil application), (2) a foliar spray with silicon dioxide nanoparticles (Si-NPs), and (3) moringa leaf extract (MLE) on the production, antioxidant defense, and physio-biochemical characteristics of spinach grown on heavy metal–contaminated soil. Bacteria and MLE or Si-NPs have been applied in single or combined treatments. It was revealed that single or combined additions significantly increased plant height, shoot dry and fresh weight, leaf area, number of leaves in the plant, photosynthetic pigments content, total soluble sugars, free proline, membrane stability index, ascorbic acid, relative water content, α-tocopherol, glycine betaine, glutathione, and antioxidant enzyme activities (i.e., peroxidase, glutathione reductase, catalase, superoxide dismutase, and ascorbate peroxidase) compared with the control treatment. However, applying bacteria or foliar spray with MLE or Si-NPs significantly decreased the content of contaminants in plant leaves (e.g., Fe, Mn, Zn, Pb, Cd, Ni, and Cu), malondialdehyde, electrolyte leakage, superoxide radical $\left(O_{2}^{\cdot -}\right)$ , and hydrogen peroxide (H_2_O_2_). Integrative additions had a more significant effect than single applications. It was suggested in our study that the integrative addition of *B. subtilis* and MLE as a soil application and as a foliar spray, respectively, is a critical approach to increasing spinach plant performance and reducing its contaminant content under contaminated soil conditions.

## 1 Introduction

Spinach (*Spinacia oleracea* L.) is a critical vegetable for the human diet, owing to its richness in carbohydrates, protein, and mineral nutrients (Oluwole et al., 2013). Spinach mainly comprises pectin, cellulose, and hemicellulose substances responsible for its firmness and texture (Sobukola et al., 2007). Spinach contains a few calories and a high percentage of salts and vitamins (Salawu et al., 2015). Each 100 g of spinach contains 2.20 g of fiber, equivalent to 10% of the amount that humans need daily (Janeczko and Timmons, 2019). Spinach also has anticancer properties because it has carotenoids (organic pigments) that give vegetables their usual colors and act as antioxidants (Salawu et al., 2015).

All metals are toxic. Nonetheless, many of them are beneficial in low concentrations. Heavy industrial activities, waste management methods, and improved farming methods have caused heavy metal pollution worldwide. The increasing dependence of agriculture on synthetic fertilizers and sewage sludge has increased the heavy metal content in terrestrial ecosystems, resulting in adverse environmental effects (Kadkhodaie et al., 2012; Kumar, 2014). Heavy metal stress, a severe abiotic stress, reduces plant production and negatively affects human and animal health and soil biodiversity (Howladar, 2014; Li et al., 2022). Upon heavy metal sensing, a complex signaling network is stimulated by the stress of heavy metal, activating specific genes to counteract this stress (Maksymiec, 2007; Li et al., 2023). Increased reactive oxygen species (ROS) production, such as hydroxyl (OH^−^), superoxide radical $\left(O_{2}^{\cdot -}\right)$ , and hydrogen peroxide (H_2_O_2_), which are highly toxic to plants, are the main consequence of heavy metal stress (Rady et al., 2019b). ROS damage DNA, lipids, proteins, and chlorophyll (Schutzendubel, 2002). Plants have an excellent antioxidant system consisting of non-enzymatic [e.g., ascorbic acid, carotenoids, tocopherols, glutathione (GSH), and proline] and enzymatic [e.g., GSH reductase (GR), peroxidase, catalase, and superoxide dismutase] antioxidants to protect them from oxidative damage (Apel and Hirt, 2004). Plants grown under heavy metal conditions are stressed in three ways: depressing water potential in soil, which causes water deficits, phytotoxicity of heavy metal ions, and imbalance of nutrients by reducing uptake or shoot transport (Marschner, 1995). Plant physiology is affected by heavy metal stress, both at the whole plant and cellular levels, through ionic and osmotic stress. It obstructs plant water relations, resulting in osmotic stress or drought physiology (Rady, 2011; Kadkhodaie et al., 2012). Heavy metal accumulation in the leaf apoplasm causes turgor loss and dehydration, leading to tissue and cell death. Among the most harshly affected processes during heavy metal stress is photosynthesis (Howladar, 2014), which is accompanied by decreased chlorophyll pigment production (Rady, 2011) and stomata closure. Consequently, the CO_2_ pressure decreases (El-Saadony et al., 2021), and rubisco is inhibited (Govarthanan et al., 2016).

The high concentration of heavy metals in ecosystems significantly affects microbial communities and activities. The toxic nature of these pollutants causes the death of many microbes, resulting in the development of specific mechanisms by other microbes that give them the ability to protect themselves from stress (Förstner and Wittmann, 2012). In bioremediation, microorganisms and plants are utilized to eliminate heavy metals. Using microorganisms is one bioremediation method. This method includes using *Nitrosomonas*, *Bacillus*, *Penicillium*, *Xanthobacter*, *Flavobacterium*, *Pseudomonas*, and *Mycobacterium* species (Qingren et al., 2001; Desoky et al., 2020; Yue et al., 2023). For instance, the isolated *Paenibacillus* sp. RM from *T. procumbens* revealed high resistance to zinc (Zn), lead (Pb), copper (Cu), and arsenic (As). Therefore, the strain *Paenibacillus* sp. RM can be used as a heavy metal bioremediator (Govarthanan et al., 2016). Desoky et al. (2020) recently used *Paenibacillus*, *Pseudomonas*, and *Bacillus* as natural biostimulants. These researchers found that these biostimulants enhanced the defense systems of spinach growing under Cd and Pb stress. On the other hand, several studies have proven that moringa leaf extract (MLE) promotes plant growth because it is rich in plant hormones, including large amounts of zeatin, carotenoids, phenols, antioxidants, osmoprotectants, and nutrients (Rady et al., 2013; Howladar, 2014; Desoky et al., 2019; Benettayeb et al., 2022). Desoky et al. (2019) reported that MLE application enhanced plant production, hormonal content, photosynthetic capacity, and antioxidative defense systems under heavy metal stress.

Silicon (Si) application enhances the ability of the plant to cope with stress (Alzahrani et al., 2018; Elrys et al., 2018a; Elrys et al., 2018b; Merwad et al., 2018; El-Saadony et al., 2021). It was revealed in former studies that Si addition adjusts nutrient imbalance, improves photosynthetic activity, and decreases heavy metal toxicity (Shi et al., 2016). Si may alter the transport and distribution of heavy metals in plant parts, thereby enhancing plant survival in soils contaminated by heavy metals (Zhang et al., 2008; Ke et al., 2022). This causes mitigation of the adverse effects of heavy metals on the photosynthetic process (Kabir et al., 2016; Jamasbi et al., 2022). Therefore, plant production could be enhanced, and heavy metal toxicity could be decreased with Si applications. However, the ameliorative effect of bulk Si sources varies genotypically among plant species and particle size (Lukačová Kuliková and Lux, 2010). Compared with bulk Si, Si nanoparticles (Si-NPs) have superior physicochemical attributes due to their microscopic size, greater surface area, reactivity, and high solubility (Qados and Moftah, 2015; Rastogi et al., 2019; El-Saadony et al., 2021). Particle size is a crucial factor affecting particle adhesion force, absorption capability, and movement into plant cells (Smith et al., 2008). Moreover, Si-NPs help plant cells in the transport of substances that can control plant metabolism and other physiological processes (Galbraith, 2007; Giraldo et al., 2014).

To our knowledge, no study has verified the co-addition of microorganisms and plant extracts or Si-NPs in relieving heavy metal stress on spinach plants growing in soil polluted with heavy metals. Our study aimed to assess the influences of (1) the soil additions of *Bacillus*, *Paenibacillus*, and *Pseudomonas*; (2) foliar spray with MLE or Si-NPs; and (3) both treatments combined in spinach grown on heavy metal–contaminated soils. Specifically, we evaluated the vegetative growth, physiological properties, antioxidant defense system, biochemical components, and heavy metal accumulation of spinach plants grown under such circumstances. We hypothesize that applying *Bacillus*, *Paenibacillus*, and *Pseudomonas* as supplements and MLE or Si-NPs as foliar spray would enhance spinach growth and production. This result would happen by adjusting ion imbalance and raising osmoprotectant contents and antioxidant activities, which play critical roles in alleviating heavy metal stress on spinach.

## 2 Materials and methods

### 2.1 Isolation, identification, and description of heavy metal–resistant bacteria

#### 2.1.1 Media and chemical used

Strains were routinely grown in a Luria Bertani (LB) medium, according to the method described by Nasiru et al. (2011), for isolation and purification.

#### 2.1.2 Soil collection

Soil samples were obtained from industrially contaminated soil from the 10th of Ramadan, Sharqia, Sharkia (longitude is 31.7423434, and latitude is 30.2926655), Egypt. Then, the soil samples were instantly brought to the laboratory.

#### 2.1.3 Isolation of heavy metal–resistant bacteria

The bacteria were isolated from the soil by blending 10 g of soil sample with 90 ml of sterilized water for 30 min using a magnetic blender. The suspension was left for 20 min. Then, 1 ml of the suspension was incubated in LB stock at 30°C at 120 rpm for 1 day. Heavy metal–resistant bacteria were selectively segregated using media containing heavy metals. A medium with a fixed concentration of 300 μg L^−1^ was preserved for each heavy metal. The segregated colonies were regrown to prepare pure cultures (Joshi and Modi, 2013).

#### 2.1.4 Estimation of minimum inhibitory concentration and tolerance to heavy metals

Minimum inhibitory concentration (MIC) was estimated using the microdilution broth method following the European Committee on Antimicrobial Susceptibility Testing. A total of 30 ml of each concentration of minerals was added to tubes containing 9 ml of Mueller Hinton broth, and 100 ml of bacteria (1.5 × 108 CFU/ml) was added to the tubes. The control treatment was composed of only Mueller Hinton broth tubes. The tubes were incubated for a day at 37°C. The MIC was the minimal mineral concentration that inhibited bacterial growth, and it was tested at 30°C for 6 days. The three bacterial strains with the highest MIC estimates for Cr, Cd, Pb, Zn, Ni, and Hg were then selected for further experimental steps (Ahirwar, 2016). The agar dilution method was used to estimate the tolerance of selected bacterial strains to different levels of heavy metals, according to Cervantes-Vega et al. (1986).

#### 2.1.5 Identification and characterization of selected bacteria

The bacteria were identified using morphological and biochemical characteristics for motility activities, following the manual of systemic bacteriology by Bergey and Holt (1989). The selected bacteria were classified on the basis of their physiological, morphological, and biochemical properties by Bille et al. (2012) following the proceedings by Logan (2009).

#### 2.1.6 Estimation of optimal bacterial growth conditions

The ideal growth conditions were assessed on the basis of temperature and pH. The selected isolates were developed in an LB agar medium at temperatures of 25°C, 30°C, 35°C, and 40°C. The pH values were 5.0, 6.0, 7.0, and 8.0. The log phase of the developed cultures under these conditions for 12–14 h was observed for the optical density at 600 nm using a spectrophotometer (Ahirwar, 2016).

### 2.2 Field experiment

#### 2.2.1 Experimental layout

A field experiment was conducted during 2021 growing season on a private farm in Abu-Zabal City, El-Qalyubia Governorate (N 30° 14′ 58″ and E 31° 21′ 16″), Egypt. The area is located within a semi-arid region, with rainfall ranging between 6 and 10 mm and daily average temperatures of 18°C ± 2°C and 12°C ± 3°C at both day and night, respectively, during the trial duration (from October to December). The relative humidity ranged between 35.4% and 38.2%, and the day length was 10–12 h. Before the settlement of the trial season, soil samples were collected randomly from the study site. The samples were preliminarily analyzed before the experiment, following the methodologies by Black (1968) and Jackson (1958) (**Table 1**). On the basis of the preliminary soil analysis, the selected soil sites were polluted by high concentrations of heavy metals (**Table 1**).

**Table 1:** Some physical and chemical properties of the investigated soil and chemical analysis of the moringa oleifera leaf extract (MLE).

Soil property		MLE component	Unit	Value
Characteristics	Value	Component		
Soil particles distribution (%)		Antioxidants and osmoprotectants		
Sand	40.65	Total free amino acids	g kg^−1^ DW	156
Silt	37.14	Free proline		132
Clay	22.21	Soluble sugars		176
Textural class	Loam	Salicylic acid	mg kg^−1^ DW	38.6
Field capacity (FC, %)		α-Tocopherol		34.4
CaCO_3_ (g kg^−1^)	13.25	Glutathione (GSH)		22.4
Organic matter (g kg^−1^)	2.87	Ascorbic acid (AsA; Vit. C)		34.8
pH*	4.62	Vitamin A (β-carotene)		163
EC (dSm^-1^) **	7.98	Selenium (Se)		0.7
Soluble cations and anions (mmolc L^−1^) **		DPPH-radical scavenging	**%**	82.2
Ca^2+^	4.76	**Phytohormones (mg kg** ^−^ **^1^ DW)**		
Mg^2+^	4.69	Total auxins		3.3
Na^+^	2.68	Total gibberellins		2.9
K^+^	2.87	Zeatin-type cytokinin		3.2
${\text{CO}}_{3}^{2-}$	0.00	**Mineral nutrients (g kg** ^−^ **^1^ DW)**		
${\text{HCO}}_{3}^{-}$	1.54	N		30.8
Cl^−^	1.95	P		15.8
${\text{SO}}_{4}^{2-}$	11.51	K		21.7
**Available nutrients (mg kg** ^−^ **^1^ soil)**		Calcium (Ca)		9.6
Nitrogen (N)	18.43	Magnesium (Mg)		4.5
Phosphorus (P)	180	Sulfur (S)		2.7
Potassium (K)	185	Fe		1.5
Lead (Pb)	321	Mn		0.8
Cadmium (Cd)	12.89			
Nickel (Ni)	113			
Iron (Fe)	4263			
Zinc (Zn)	252			
Manganese (Mn)	102			
Copper (Cu)	787			

*Soil suspension 1:2.5.

**Soil paste extract.

Healthy spinach (*Spinacia Oleracea* L., c.v. Polar bear R Z, F1-Hybrid) seeds were brought from the Vegetative Research Section, Horticulture Research Institute, Agricultural Research Centre, Giza. Before sowing the seeds on 25 October 2021, ammonium sulfate at 280 kg ha^−1^ (20% N) and potassium sulfate at 200 kg ha^−1^ (48.5% K_2_O) were broadcast-applied. The experiment followed the split plot design with bacteria treatments as main plots and the foliar amendments as subplots. The treatments were triplicated. After washing with distilled water, the seeds were sterilized with NaClO for 2 min, cleaned out by distilled water, and then left to dry at room temperature (25°C). After air drying, the seeds were sown at a rate of 15 kg ha^−1^ in hills (spaced 15–20 cm) with spacing of 60 cm between rows in plots with an area of 10.5 m^2^ for each (3.0 m long × 3.50 m width). Four seeds per each hill were used, which were thinned later to two seedlings before the first irrigation using a drip irrigation system.

#### 2.2.2 Application of selected bacteria

The selected bacterial strains (SA6, AT26, and MM40) were grown on T- medium Duxbury (1981) with 1.0 × 10^8^ to 1.0 × 10^9^ CFU L^−1^ as the viable number determined by the plate counts method of the bacterial broth culture. Bacterial strains were added at a rate of 10 L ha^−1^ as a soil application in the irrigation water (second, fourth, and sixth irrigations), at three equal doses at 15, 30, and 45 days after sowing. The bacterial strains were applied during the last 10 min of drip irrigation.

#### 2.2.3 Preparation and application of MLE and Nano-SiO_2_ (Si-NPs)

The *Moringa oleifera* leaves were mixed with 80% ethanol at a rate of 200 g/2 L and stirred using a homogenizer as recommended by Makkar and Becker (1996). The *Moringa oleifera* leaf extract was filtered out using Whatman No. 2 filter paper, which was within 5 h from extraction process, or stored at −20°C until use. The used nano-silicon dioxide (99.5% pure; 20–30 nm) (Sigma-Aldrich Chemie GmbH, Taufkirchen, Germany) was 180–600 m^2^ g^−1^ as the surface area. MLE and Si-NPs were applied as foliar spray at rates of 5 ml L^−1^ and 1.5 mM nano-SiO_2_, respectively, at three times during the experiment, 15, 30, and 45 days after sowing. MLE and Si-NPs were sprayed using a pressurized spray bottle (0.1% Tween 20 as a surface spreader). Foliar application of distilled water was set as a control.

#### 2.2.4 Vegetative growth and yield components

The plant height for 10 spinach plants in each treatment was estimated after cutting the plants in addition to plant height, leaf area, number of leaves, and fresh and dry weight of shoot (grams per plant). The shoot was dried in an oven at 70°C until constant weight.

#### 2.2.5 Determinations of physicochemical properties

Photosynthetic pigment contents (total chlorophylls and carotenoids) were determined using the acetone extract method proposed by Fadeel (1962). Absorbance readings were recorded at 480, 645, and 663 nm using a spectrophotometer (Beckman 640D, USA) to calculate the contents of pigments in mg g^−1^ leaf fresh weight (FW). The membrane stability index (MSI) was determined using 200 mg of fresh leaf (two sets) in test tubes containing 10 cm^3^ of double-distilled water. One group of samples was heated at 40°C for 30 min. EC was recorded on a conductivity bridge (C1). The second group of samples was boiled at 100°C for 10 min in a boiling water bath, and Electrical conductivity (EC) was measured (C2). As in the study by Premachandra et al. (1990), modified by Rady (2011), the MSI was calculated using the following formula: MSI (%) = (1 − [C1/C2]) × 100.

The method by Barrs and Weatherley (1962) was used to estimate the relative water content (RWC). The FW of the leaves was measured, and the leaves were left drenched in water for 3 h. Then, the turgid weight (TW) of the leaves was calculated. The samples were then dried in an oven at 80°C for 24 h and weighed (DW). The RWC was determined using the following formula: RWC = [(FW − DW)/(TW − DW)] × 100.

The malondialdehyde (MDA) content (μmol g^−1^ FW) was measured in 0.1 g leaf homogenized in a Na-phosphate buffer. The homogenate was centrifuged under cooling at 20,000 × g for 25 min. The supernatant was read at 532 nm and corrected for non-specific turbidity at 600 nm, following the methods proposed by Heath and Packer (1968).

Electrolyte leakage (EL) was measured in a solution of 20 leaf discs 0.5 cm subjected to room temperature, 45°C–55°C for 30 min, and 100°C for 10 min to measure ECa, ECb, and ECc (electrical conductivity), respectively. EL was calculated using the following formula, as proposed by Sullivan (1979): {EL (%) = [(ECb − ECa)/ECc] × 100}.

The superoxide $\left(O_{2}^{-}\right)$ content (µmol g^−1^ FW) was determined by immersing leaf fragments in 10 mM K-phosphate buffer (pH 7.8), 0.05% Nitro blue tetrazolium chloride (NBT), and 10 mM NaN_3_ for 1 h. After heating the immersed solution (at 85°C for 15 min) and rapidly cooling, the absorbance was read at 580 nm (Kubiś, 2008).

The H_2_O_2_ content (µmol g^−1^ FW) was determined in the acetone extract. After adding titanium reagent and ammonium, the extract was dissolved in 1 M H_2_SO_4_, and the absorbance was measured at 415 nm (Mukherjee and Choudhuri, 1983). The dried powdered spinach leaves were weighed to estimate their contents of heavy metals, which were determined using atomic absorption spectrophotometry according to AOAC (1984).

#### 2.2.6 Determination of non-enzymatic antioxidant compounds

The content of α-tocopherol (α-TOC) in the dried leaves in micromoles per gram was determined by a high-performance liquid chromatography system with a mobile phase (94 ml of methanol and 6 ml of water) and a 1.5 ml/min flow rate at 292 nm of a UV detector (Konings et al., 1996; Ching and Mohamed, 2001). Nearly 0.02 g of butylated hydroxyl toluene was added to 0.9 L of extraction solvent (n-hexane-ethyl acetate, n-hexane + 0.1 L of CH_3_–COO–CH_2_–CH_3_). The standard solution and dilutions of 0.02–0.2 mg/ml were prepared using R-TOC (0.05 g/0.1 L of n-hexane). The ascorbic acid (AsA) level (μmol/g of leaf FW) was determined following the method by Kampfenkel et al. (1995). The GSH level (μmol/g of leaf FW) was determined according to Griffith (1980). Soluble sugars were extracted using 96% v/v ethanol, interacted with 3 ml of freshly prepared reagent [150 mg + H_2_SO_4_ (100 ml, 72% v/v)], boiled for 10 min in a water bath, and cooled. Then, the contents (mg/g of leaf DW) were measured using a spectronic spectrophotometer (Bausch and Lomb-2000) at 625 nm (Irigoyen et al., 1992). The proline content (μmol g^−1^ of leaf DW) was determined using standard L-proline prepared according to Bates et al. (1973). Glycine betaine levels were measured following the methods by Di Martino et al. (2003).

#### 2.2.7 Estimation of antioxidant activities

To obtain enzyme extraction, gently cleaned 0.5 g of fresh leaf was homogenized in ice-cold 0.1 M phosphate buffer (pH 7.5) containing 0.5 mM EDTA (Vitória et al., 2001). Under cooling, the homogenate was then centrifuged at 15,000 × *g* for 15 min. The supernatant was then referred to as the enzyme extract. The activity of catalase (CAT) was assayed spectrophotochemically according to Chance and Maehly (1955). The enzyme extract (100 µl) was added to 100 µl of 100 mM H_2_O_2_, and the total volume was made up of 1 ml of 250 mM phosphate buffer (pH 6.8). The decrease in the optical density at 240 nm against blank was recorded every minute. Ascorbate peroxidase (APX) was assayed spectrophotochemically according to Fielding and Hall (1978). The assay was carried out at 25°C in a 1.0-cm light path cuvette, and the reaction mixture consisted of 1,500 µl of phosphate buffer ((pH 7.0), 20 µl of EDTA, 1,000 µl of sodium ascorbate, and 20 µl of enzyme extract. After mixing, the reaction was initiated by adding 480 µl of H_2_O_2_, and the decrease in the optical density at 290 nm against blank (without extract) was continuously recorded every minute (for 2 min). Thomas et al. (1982) method was used to estimate the activity of peroxidase (POD) in spinach leaves. The enzyme was assayed using guaiacol as the substrate. The reaction mixture consisted of 3 ml of phosphate buffer (0.1 M, pH 7.0), 30 µl of H_2_O_2_ (20 mM), 50 µl of enzyme extract, and 50 µl of guaiacol (20 mM). The reaction mixture was incubated in a cuvette for 10 min at room temperature. The optical density was measured at 436 nm, and the enzyme activity was expressed as the number of absorbance units g^−1^ FW of leaves. Superoxide dimutase (SOD) activity was measured on the basis of the absorbance peak of superoxide–nitro blue tetrazolium complex (Sairam et al., 2002). The activity of GR was estimated by recording three absorbances at 340 nm after detecting the oxidation of NADPH for Rao et al. (1996).

#### 2.2.8 Statistical analysis

The significance of differences between the means of variables was statistically compared at *p* ≤ 0.05 using Duncan’s multiple range test. COSTAT computer software (CoHort Software version 6.303, Berkeley, CA, USA) was used to carry out the statistical analysis.

## 3 Results and discussions

### 3.1 *In vitro* assay

#### 3.1.1. Isolation of heavy metal resistant bacteria

In this study, 135 bacterial colonies were examined using an LB agar medium with heavy metals to identify, isolate, and characterize resistant bacteria in polluted industrial soils. Among the analyzed colonies, 61 isolates were chosen for a secondary examination. Later, three strains were selected according to their resistance to heavy metals and were further utilized in this study. *B. subtilis* (SA6) and *Paenibacillus* (AT26) strains were gram-positive, rod-shaped motile bacteria. The *Pseudomonas aeruginosa* (MM40) strain was gram-negative and rod-shaped. These isolates had optimum growth at 35°C ± 2°C and pH 7.0.

#### 3.1.2 Growth studies of bacteria and simultaneously resistant to heavy metals

Growth studies of the strains SA6, AT26, and MM40 were conducted in an LB medium with Zn, Ni, Pb, Cd, Ag, Hg, and Cr (200 mg L^−1^) prepared from stock solutions. The measurements from the cultures incubated for 48 h agreed with bacterial resistance for every heavy metal. All isolates were sensitive to Ag, Hg, and Ni. Consequently, microorganisms with simultaneous resistance to heavy metals accompanied by their biotransformation into non-toxic forms would be potentially helpful for the detoxification of soils contaminated by heavy metals.

#### 3.1.3 MIC of heavy metal

Soil bacteria from strains SA6, AT26, and MM40 had high resistance to all heavy metals. MIC values varied from 0.82 to 12.9 mM. Among the heavy metals, Ni and Zn were less toxic, whereas Hg and Cr were highly toxic to all strains. The MIC values of heavy metals are shown in **Table 2**.

**Table 2:** Minimum inhibitory concentration (MIC) of heavy metal for soil bacterial isolates.

S. No.	Heavy metals	Isolated bacterial colonies		
		*Bacillus subtilis* ssp. *spizizenii*	*Paenibacillus jamilae*	*Pseudomonas aeruginosa*
1	Zinc	4.6	12.9	8.8
2	Nickel	2.1	3.2	7.2
3	Lead	3.9	4.9	5.1
4	Cadmium	5.6	7.1	4.5
5	Silver	1.5	2.2	1.4
6	Mercury	1.4	1.9	0.82
7	Chromium	5.5	7.5	4.1

Heavy metal concentration in micromolars.

#### 3.1.4 Identification of the bacterial isolates according to morphological and biochemical characteristics

##### 3.1.4.1 Checking and identification of utilized bacterium

Initially, only three bacterial isolates were selected from the 18 isolates collected from the contaminated soil sample. A total of 200 µg ml^−1^ of heavy metals was used when choosing the first concentration (first screening). The bacterial isolates displayed a high ability of heavy metal resistance while we screened these isolates utilizing the second concentration (heavy metals at 300 µg/ml in the second screening). Primary identification for the three bacterial isolates was made on the basis of 36 tests, including gram reaction, motility, cell morphology, oxygen requirement, spore formation, and biochemical and physiological tests for *Bacillus*, *Paenibacillus*, and *Pseudomonas* identifications (Brenner et al., 2005; Logan, 2009; Priest, 2009). Considering the obtained results and after careful observation and comparison of the recovered morphological, physiological, and biochemical characteristics of the four screened bacteria, they were compared with known taxa in Bergey’s Manuals. Therefore, it could be inferred that the four screened bacteria, coded with SA6, AT26, and MM40, were identified at the genus and species levels as *B. subtilis* ssp*. spizizenii*, *P. jamilae*, and *P. aeruginosa*, respectively.

As for *Bacillus* species, one bacterial isolate of the three bacterial isolates were linked with *Bacillus* genus, which is located in Phylum XIII: Firmicutes, Class I: Bacilli, Order: Bacillales, and Family: Bacillaceae. The great ratios of *Bacillus* spp. isolates may be due to their common strains’ presence anywhere, cultivation in the used media, or favorite various ecosystems for their growth (Zhang et al., 2010).

##### 3.1.4.2 Direct identification of the most active bacteria synthesizing silver nanoparticles using MALDI-TOF-MS

Matrix-assisted laser desorption produces a spectrum when a protein of an intact microorganism is directly detected, providing rapid identification (Claydon et al., 1996; Holland et al., 1996). Concerning the speed of bacterial identification, Seng et al. (2009) counted 6 min as the base identification time by MALDI-TOF-MS, whereas traditional techniques recorded 48 h for the same identification. Cherkaoui et al. (2010) observed that the analyses of co-isolates could be complemented in a shortened processing time (<15 min) by MALDI-TOF-MS.

The bacterial strains investigated in this study were included in the Bruker Database, with spectrum scores above 2.0. Therefore, the bacterial biosynthesis of Ag nanoparticles was accurately detected using a Micro Flex LT mass spectrophotometer, with a scoring rate higher than 2.0 of the biotype software used, agreeing well with Moussaoui et al. (2010) and Stevenson et al. (2010). Moreover, Bille et al. (2012) indicated that this method properly classified bacteria cultivated in solid media with a precision of 99.2%. However, only one strain in some cases was identified at the genera, species, and subspecies levels, as in isolate no. 6 (SA6) (**Table S1**). Thus, these isolates were fully identified as *B. subtilis* ssp. *spizizenii* DSM 15029T DSM with a similarity score of 2.236 (**Table S1**).

In addition, the results in **Table S1** were not significantly different from the results of the identification obtained with the Bruker Daltonies MALDI-TOF-MS instrument or other conventional techniques, such as biochemical- and morphological-based methods. This result is in agreement with the identification results of 16S rRNA sequencing by the MALDI-TOF-MS (Bizzini et al., 2010; Zhang et al., 2010) due to the coevolution of ribosomal proteins and nucleic acids (Teramoto et al., 2009; Sauer and Kliem, 2010).

### 3.2 Spinach (Spinacia oleracea L.) plants as a bioassay parameter in bio-removal of heavy metals and or foliar spray with MLE or Si-NPs

#### 3.2.1 Vegetative growth parameters

Compared with the control treatment, single amendments [i.e., *B. subtilis* (SA6), *P. jamilae* (AT2), and *P. aeruginosa* (MM40) added as soil supplementation or MLE and Si-NPs applied as foliar spray)] or integrative treatments (bacteria soil application + MLE or Si-NPs) significantly increased plant height, leaf area, leaf number, shoot fresh, and dry weight of spinach plants grown on heavy metal–contaminated soil (**Table 3**). Integrative applications of amendments outperformed single treatments. The application of *B. subtilis* accompanied by MLE applied as the foliar spray was the best treatment compared with other combined applications. This treatment increased plant height by 193%, leaf area by 197%, leaf number by 223%, shoot FW by 273%, and shoot DW by 171%. This increase was significant compared with the control plants grown under heavy metal stress without bacterial application or foliar spray.

**Table 3:** Changes in growth attributes of heavy metal–stressed spinach plant in response to bioremediation of heavy metals bacterial applied as soil inoculation and/or foliar spray with MLE or Si-NPs.

		Plant height (cm)	Leaf area (cm^2^)	No. leaf plant^-1^	Fresh weight (Mg ha^−1^)	Dry weight (Mg ha^−1^)
Effect of bacterial soil application						
Without		11.4 ± 0.25^d^	34.8 ± 1.6^d^	6.55 ± 0.11^d^	11.4 ± 0.25^d^	0.95 ± 0.07^d^
Bs		26.2 ± 1.1^a^	94.4 ± 2.3^a^	16.4 ± 0.15^a^	34.4 ± 1.3^a^	2.64 ± 0.11^a^
Pj		22.1 ± 1.3^b^	74.3 ± 2.9^b^	14.2 ± 0.16^b^	27.8 ± 1.2^b^	2.07 ± 0.12^b^
Psa		16.2 ± 1.3^c^	49.5 ± 1.8^c^	11.0 ± 0.13^c^	17.8 ± 0.66^c^	1.47 ± 0.09^c^
**Effect of foliar spray**						
Without		17.1 ± 1.1^c^	60.1 ± 3.1^c^	10.8 ± 0.12^c^	20.7 ± 0.75^c^	1.49 ± 0.11^a^
Si-NPs		19.1 ± 1.3^b^	62.9 ± 3.6^b^	12.0 ± 0.14^b^	22.67 ± 0.63^b^	1.73 ± 0.13^b^
MLE		20.7 ± 1.4^a^	66.4 ± 3.8^a^	13.1 ± 0.17^a^	25.3 ± 0.55^a^	1.87 ± 0.14^a^
**Effect of interaction**						
**Soil application**	**Foliar spray**					
Without	Without	9.65 ± 0.36^k^	32.5 ± 1.1^l^	5.34 ± 0.16^j^	9.84 ± 0.21^k^	0.95 ± 0.08^f^
	Si-NPs	11.5 ± 0.33^j^	34.6 ± 1.3^k^	6.67 ± 0.15^i^	11.3 ± 0.33^j^	0.097 ± 0.06^f^
	MLE	13.4 ± 0.41^i^	37.3 ± 1.5^j^	7.68 ± 0.17^h^	13.2 ± 0.25^i^	1.05 ± 0.06^f^
Bs	Without	17.6 ± 0.52^f^	54.6 ± 2.1^g^	12.4 ± 0.12^e^	19.6 ± 0.42^f^	1.56 ± 0.10^de^
	Si-NPs	26.4 ± 1.4^b^	94.7 ± 3.2^b^	16.4 ± 0.15^b^	34.1 ± 0.36^b^	2.38 ± 0.15^b^
	MLE	28.3 ± 1.5^a^	96.7 ± 3.8^a^	17.3 ± 0.17^a^	36.8 ± 96^a^	2.58 ± 0.19^a^
Pj	Without	16.3 ± 0.66^g^	48.3 ± 1.6^h^	11.0 ± 0.10^f^	17.5 ± 0.57^g^	1.51 ± 0.11^e^
	Si-NPs	23.7 ± 1.4^c^	77.1 ± 2.8^d^	15.3 ± 0.19^c^	31.6 ± 1.3^c^	2.04 ± 0.18^bc^
	MLE	24.0 ± 1.6^c^	91.5 ± 3.1^c^	15.4 ± 0.21^c^	32.3 ± 1.6^c^	2.31 ± 0.13^ab^
Psa	Without	14.5 ± 0.74^h^	44.9 ± 2.2^i^	9.67 ± 0.31^g^	16.3 ± 0.66^h^	1.33 ± 0.08^e^
	Si-NPs	20.4 ± 1.7^e^	71.5 ± 1.6^f^	13.0 ± 0.33^e^	24.3 ± 1.7^e^	1.82 ± 0.09^cd^
	MLE	22.3 ± 1.2^d^	74.2 ± 2.2^e^	14.5 ± 0.26^d^	27.7 ± 1.8^d^	1.87 ± 0.06^c^

Data are means (n = 9) ± SE. The same letters in each column indicate no significant differences according to the LSD test (p ≤ 0.05). Without, treated with tap water; Bs, Bacillus subtilis ssp; Pj, Paenibacillus jamilae; Psa, Pseudomonas aeruginosa; Si-NPs, silicon dioxide nanoparticles; MLE, moringa leaf extract.

Rady and Hemida (2015) reported reductions in growth and biomass under heavy metal stress on spinach plants, indicating low resistance to heavy metal stresses. This decreased heavy metal tolerance can be linked to the uptake of heavy metals by roots and rapid translocation to shoots in toxic quantities that impact physiological and biochemical processes in plant cells. In turn, this process reduces plant vigor, inhibits plant growth, and stimulates oxidative damage of cellular constituents (Kopittke et al., 2010; Kong et al., 2023). Heavy metal stress inhibits division rates and cell elongation by barring the proton pump. As a result, there is a reduction in plant growth and limited yield, with a decrease in the biomass production of the plant (Choudhury and Panda, 2004). Heavy metal stress alters plant growth and cell division in the meristematic zone of root and shoot systems (Kang et al., 2009; Kaur et al., 2012).

Living cells can promote biomass building and plant growth, mitigating and repairing the damage caused by ROS overproduction in response to stress caused by the heavy metals available in the plant-growing medium. Several microorganisms in the rhizosphere of plants, such as mycorrhizal fungi, can increase their capability to uptake heavy metals (Bojórquez et al., 2016). Joner and Leyval (1997) reported increments of 90%, 127%, and 131% in the Cd uptake by mycorrhizal plants compared with non-mycorrhizal soil with Cd concentrations of 1, 10, and 100 mg kg^−1^, respectively. Microbial treatment of soil contamination with heavy metals adheres to considerable advantages, including conservation of the soil structure and low costs (Jovancicevic et al., 1987). Numerous species, such as fungi and bacteria [i.e., *Pseudomonas* (Vullo et al., 2008) and *Bacillus* (Wierzba, 2015)], have significant remediation capacity. We have shown that several bacteria can attract or release heavy metals within the soil and promote plant growth and biomass formation. These improvements in the development and total biomass of the growing plants under heavy metal stress may be due to the diminished endogenous levels of heavy metal ions by bacteria living cells.

On the other hand, it was shown in the analysis of MLE that this extract can be utilized as a plant biostimulant. This extract contains essential micro and macro elements, AsA, antioxidants (e.g., proline), phytohormone [e.g., gibberellins and indol-3-acetic acid (IAA)], cytokinin (e.g., zeatin), and total soluble sugar (Howladar, 2014; Desoky et al., 2018; Desoky et al., 2019; Ramzan et al., 2022). It was shown in this study that applying MLE improved growth parameters due to the stimulation and mobilization of metabolites/inorganic solutes (e.g., zeatin, IAA, Ca, and K), which are present in MLE, to growing plumule. These solutes can enhance germination by stimulating amylase enzyme activity, decreasing sugar from water absorption, and boosting cell elongation (Afzal et al., 2012). Si-NPs also significantly promoted plant growth due to their beneficial impacts on plant nutrition and mechanical strength and, thus, plant resistance to abiotic stresses (Gao et al., 2006; Hamayun et al., 2010; Liu et al., 2014). In this study, Si-NPs alleviated the effects of heavy metal stress in spinach plants, enhancing growth due to the improvement of the photosynthesis rate, ribulose biphosphate carboxylase activity, and chlorophyll leaf content (Hamayun et al., 2010; Xu et al., 2022).

#### 3.2.2 Photosynthetic pigments, relative water content, and membrane stability index

Chlorophyll a and b and carotenoids, along with the RWC and the MSI of spinach plants grown on heavy metal–contaminated soils, had significant responses to both single and integrative amendments compared with the control (without bacteria application + without foliar spray) (**Table 4**). Moreover, the effects of the integrative amendments significantly exceeded those of the single treatments. The integrative *B. subtilis* applied as soil application in integration with MLE used as foliar spray resulted in increments in chlorophyll a (98.6%), chlorophyll b (32.4%), carotenoids (103%), RWC (54.6%), and MSI (42.5%).

**Table 4:** Changes in photosynthetic pigments, relative water content (RWC), and membrane stability index (MSI) of heavy metal–stressed spinach plant in response to bioremediation of heavy metals bacterial applied as soil inoculation and/or foliar spray with MLE or Si-NPs.

		Ch a(mg g^−1^)	Ch b(mg g^−1^)	Carotenoids (mg g^−1^)	RWC(%)	MSI(%)
**Effect of bacterial soil application**						
Without		1.71 ± 0.11^d^	0.54 ± 0.02^d^	0.89 ± 0.06^d^	60.5 ± 2.1^d^	55.4 ± 1.6^d^
Bs		2.60 ± 0.16^a^	0.87 ± 0.04^a^	1.42 ± 0.09^a^	83.5 ± 3.6^a^	69.5 ± 1.8^a^
Pj		2.45 ± 0.14^b^	0.80 ± 0.05^b^	1.34 ± 0.11^b^	78.5 ± 3.2^b^	66.3 ± 2.6^b^
Psa		2.27 ± 0.13^c^	0.72 ± 0.03^c^	1.22 ± 0.12^c^	72.9 ± 3.9^c^	63.6 ± 2.8^c^
**Effect of foliar spray**						
Without		1.89 ± 0.13^c^	0.61 ± 0.02^c^	1.03 ± 0.11^c^	65.1 ± 2.8^c^	57.7 ± 3.2^c^
Si-NPs		2.41 ± 0.15^b^	0.78 ± 0.06^b^	1.29 ± 0.09^b^	77.1 ± 3.1^b^	66.2 ± 3.3^b^
MLE		2.48 ± 0.16^a^	0.81 ± 0.05^a^	1.33 ± 0.13^a^	79.2 ± 3.5^a^	67.1 ± 3.4^a^
**Effect of interaction**						
**Soil application**	**Foliar spray**					
Without	Without	1.50 ± 0.11^i^	0.74 ± 0.02^k^	0.76 ± 0.06^l^	58.7 ± 2.8^l^	52.4 ± 2.8^j^
	Si-NPs	1.78 ± 0.13^h^	0.57 ± 0.03^j^	0.94 ± 0.08^k^	60.5 ± 2.3^k^	56.5 ± 2.6^i^
	MLE	1.86 ± 0.15^g^	0.58 ± 0.4^j^	0.98 ± 0.05^j^	62.4 ± 2.7^j^	57.2 ± 2.7^h^
Bs	Without	2.12 ± 0.21^e^	0.69 ± 0.02^g^	1.17 ± 0.09^g^	69.5 ± 2.7^g^	60.5 ± 2.9^f^
	Si-NPs	2.79 ± 0.23^b^	0.94 ± 0.06^b^	1.53 ± 0.11^b^	89.7 ± 2.9^b^	73.6 ± 3.6^b^
	MLE	2.98 ± 0.25^a^	0.98 ± 0.07^a^	1.55 ± 0.13^a^	90.8 ± 2.6^a^	74.7 ± 3.4^a^
Pj	Without	2.03 ± 0.23^f^	0.66 ± 0.03^h^	1.12 ± 0.12^h^	67.1 ± 3.4^h^	59.7 ± 2.5^f^
	Si-NPs	2.64 ± 0.26^c^	0.86 ± 0.02^d^	1.43 ± 0.13^d^	83.2 ± 3.5^d^	69.3 ± 3.4^c^
	MLE	2.69 ± 0.27^c^	0.89 ± 0.05^c^	1.47 ± 0.13^c^	85.3 ± 3.6^c^	69.8 ± 3.7^c^
Psa	Without	1.92 ± 0.13^g^	0.62 ± 0.06^i^	1.05 ± 0.08^i^	65.1 ± 3.7^i^	58.4 ± 2.1^g^
	Si-NPs	2.42 ± 0.29^d^	0.77 ± 0.04^f^	1.28 ± 0.06^f^	75.1 ± 2.9^f^	65.6 ± 2.5^d^
	MLE	2.47 ± 0.24^d^	0.79 ± 0.06^e^	1.32 ± 0.07^e^	78.6 ± 3.6^e^	66.8 ± 3.8^d^

Data are means (n = 9) ± SE. The same letters in each column indicate no significant differences according to the LSD test (p ≤ 0.05). Without, treated with tap water; Bs, Bacillus subtilis ssp.; Pj, Paenibacillus jamilae; Psa, Pseudomonas aeruginosa; Si-NPs, silicon dioxide nanoparticles; MLE; moringa leaf extract.

Heavy metal toxicity in the planting medium causes excess toxic impacts on plants, such as the induction of leaf chlorosis (Wierzbicka, 1994). This results in the surplus production of ROS, which stimulates oxidative stress (Cheeseman, 2007). Consequently, there is an increase in ROS damage to chlorophylls, membrane functions, DNA, and proteins. Heavy metal toxicity causes osmotic issues (physiological drought), oxidative stresses, and ion disequilibrium (Zhu, 2001; Lang et al., 2023), preventing the synthesis of photosynthetic processes and pigments (Sheng et al., 2008).

In addition, bean plants treated with heavy metals significantly decreased the chlorophyll content (Howladar, 2014). This decrease may be due to the increased activity of chlorophyll-degraded enzyme chlorophyllase under heavy metal stress conditions (Reddy and Vora, 1986). The photosynthesis, RWC, and MSI values were significantly lower in the heavy metal–stressed spinach plants, which agrees with the findings observed by Semida et al. (Semida et al., 2018). However, the activity of the antioxidant defense systems was notably higher than that of the control. This could be linked to the basic function of antioxidants in decreasing the stress response of plants to higher concentrations of heavy metals (Singh et al., 2016). Plant processes related to photosynthesis, chlorosis, growth, water absorption, and nutrient uptake are altered if plants are exposed to high levels of heavy metals, eventually leading to death (Agami and Mohamed, 2013). Applying heavy metal bacteria changes the decline in chlorophyll content and water consumption under heavy metal toxicity. Bacteria have a key mechanism *via* connecting heavy metals to the cell walls and prohibiting their movement to plants. This reduces the pervasive effects of heavy metals on plants, counteracts premature leaf senescence, and increases leaf area and photosynthesis (Cervantes et al., 2001). Many microorganisms may limit the accumulation of heavy metals in plants, causing bioavailability and transfer rates of heavy metals within the rhizosphere to roots by apoplastic or symplastic pathways.

Moreover, heavy metal uptake can be affected by the cation-exchangeable capacity (CEC) of the cell walls in the root system. A high CEC indicates more metal adsorption by the cell walls, which causes more available metal ions for the membrane transfer. In addition, high CEC can amplify the metal content in the cell cytoplasm and thus impact metal tolerance in the plant (Nazar et al., 2012). Remediation with heavy metal–resistant bacteria decreases the toxicity of heavy metals. This process affects several developmental processes, including antioxidative mechanisms concerning the defense against purine breakdown. Finally, metabolism processes that increase photosynthetic pigments, gas exchange, and water statements may be alleviated (Cervantes et al., 2001).

On the other hand, MLE contains Fe, which activates enzymes involved in the chlorophyll biosynthesis pathway, and some antioxidant enzymes, such as APOX and GSH, which release ROS and protect chlorophyll from degradation (Zayed et al., 2011; Nwagbara et al., 2022). The major controlling factor of photosynthesis depends on the role of K in stomatal regulation (Brugnoli and Bjo¨Rkman, 1992). The increase in growth characteristics and chlorophyll content of spinach plants grown under heavy metal stress is reflected in the increasing shoot system. This feature might be attributed to high assimilation, which correlates with macro- and micronutrients, gibberellin, and selenium (Yildirim et al., 2009; Nikee et al., 2014). At the same time, the increase in chlorophyll may be attributed to MLE, preventing premature leaf senescence and resulting in more leaf area, which increases photosynthetic pigments (Rehman and Basra, 2010). The application of MLE can improve the chlorophyll content owing to altering leaf senescence by its contents of mineral nutrients, phytohormones, and antioxidants (Elzaawely et al., 2016). Si-NPs also improved the photosynthetic pigments of spinach plants grown under heavy metal conditions. Ming et al. (2011) indicated that such photosynthesis improvement could be linked to mitigating stress-induced damage. The content of organic compounds (e.g., proteins, chlorophyll, and phenols) notably improved with the application of Si-NPs in maize plants (Suriyaprabha et al., 2012). Similarly, Haghighi and Pessarakli (2013) indicated that Si-NPs improved photosynthesis due to stomata changes rather than by stimulating the chlorophyll content. The water content of plants under salinity stress may be increased by Si owing to decreasing the osmotic potential and augmenting the turgor pressure (Romero-Aranda et al., 2006; Eissa et al., 2022; Sharma et al., 2022).

#### 3.2.3 Oxidative stress

Compared with the control treatment, EL, MDA, H_2_O_2_, and $O_{2}^{-}$ decreased in plants from all remediation treatments in which heavy metal–resistant bacteria were used as a soil application or foliar spray was applied with Si-NPs or MLE (**Table 5**). Among all the integrative treatments, the addition of *B. subtilis* as a soil application integrated with MLE as the foliar spray was the best treatment. In that treatment, MDA decreased by 74.1%, EL by 57.8%, $O_{2}^{-}$ by 60.6%, and H_2_O_2_ by 73.5% compared with the control treatment plants.

**Table 5:** Changes in malondialdehyde (MDA), electrolyte leakage (EL), hydrogen peroxide (H_2_O_2_), and superoxide radical $\left(O_{2}^{\cdot -}\right)$ of heavy metal–stressed spinach plant in response to bioremediation of heavy metals bacterial applied as soil inoculation and/or foliar spray with MLE or Si-NPs.

		MDA(µmol g^−1^ FW)	EL(%)	$O_{2}^{\cdot -}$ µmol g^−1^ FW)	H_2_O_2_(µmol g^−1^ FW)
Effect of bacterial soil application					
Without		3.34 ± 0.15^a^	14.7 ± 0.65^a^	0.801 ± 0.06^a^	15.8 ± 0.85^a^
Bs		1.52 ± 0.11^d^	8.50 ± 0.23^d^	0.462 ± 0.03^b^	7.22 ± 0.32^d^
Pj		1.82 ± 0.12^c^	9.15 ± 0.33^c^	0.532 ± 0.04^c^	8.46 ± 0.45^c^
Psa		2.23 ± 0.16^b^	10.3 ± 0.85^b^	0.361 ± 0.02^b^	10.1 ± 0.74^b^
**Effect of foliar spray**					
Without		2.98 ± 0.18^a^	13.1 ± 0.96^a^	0.731 ± 0.04^a^	14.1 ± 0.85^a^
Si-NPs		1.92 ± 0.13^b^	9.88 ± 0.56^b^	0.566 ± 0.03^b^	8.87 ± 0.58^b^
MLE		1.78 ± 0.11^c^	9.10 ± 0.85^c^	0.521 ± 0.04^c^	8.25 ± 0.67^c^
**Effect of interaction**					
**Soil application**	**Foliar spray**				
Without	Without	3.54 ± 0.18^a^	16.3 ± 0.98^a^	0.880 ± 0.8^a^	17.6 ± 1.2^a^
	Si-NPs	3.32 ± 0.16^b^	14.7 ± 0.86^b^	0.776 ± 0.06^b^	15.5 ± 1.1^b^
	MLE	3.15 ± 0.18^c^	13.2 ± 0.76^c^	0.746 ± 0.05^c^	14.4 ± 1.3^c^
Bs	Without	2.65 ± 0.12^f^	11.3 ± 0.74^f^	0.646 ± 0.04^e^	11.9 ± 0.96^e^
	Si-NPs	1.01 ± .08^k^	7.31 ± 0.68^j^	0.393 ± 0.01^j^	5.03 ± 0.32^h^
	MLE	0.92 ± 0.03^l^	6.87 ± 0.32^k^	0.346 ± 0.02^k^	4.66 ± 0.11^h^
Pj	Without	2.80 ± 0.16^e^	11.9 ± 0.25^e^	0.683 ± 0.04^d^	13.1 ± 0.96^d^
	Si-NPs	1.38 ± 0.12^i^	7.98 ± 0.66^i^	0.486 ± 0.03^h^	6.46 ± 0.52^g^
	MLE	1.29 ± 0.06^j^	7.57 ± 0.74^j^	0.426 ± 0.04^i^	5.83 ± 0.32^g^
Psa	Without	2.94 ± 0.14^d^	12.6 ± 0.99^d^	0.716 ± 0.06^c^	13.7 ± 0.42^d^
	Si-NPs	1.99 ± 0.09^g^	9.53 ± 0.89^g^	0.610 ± 0.05^f^	8.50 ± .048^f^
	MLE	1.76 ± 0.08^h^	8.74 ± 0.75^h^	0.566 ± 0.03^g^	8.03 ± 0.47^f^

Data are means (n = 9) ± SE. The same letters in each column indicate no significant differences according to the LSD test (p ≤ 0.05). Without, treated with tap water; Bs, Bacillus subtilis ssp.; Pj, Paenibacillus jamilae; Psa, Pseudomonas aeruginosa; Si-NPs, silicon dioxide nanoparticles; MLE, moringa leaf extract.

As an indicator of lipid peroxidation, the MDA content is an excellent biochemical parameter of the stress tolerance/sensitivity (Hernández and Almansa, 2002). MDA was linked with oxidative stress ( $O_{2}^{-}$ and H_2_O_2_) and raised in spinach plants under heavy metal stress (**Table 5**). The increments in MDA and oxidative stress are linked to increased EL and decreased cellular water content and membrane integrity, which affect metabolic functions and, subsequently, plant biomass production (Rady et al., 2019a). The acceptance of electrons by NADP will be reduced by using oxygen as an electron receptor, producing more ROS, such as ^1^O_2_, $O_{2}^{-}$ , H_2_O_2_, and OH^−^ radicals, resulting in cell membrane peroxidation (Nikee et al., 2014). However, stress spinach treated with remediation of heavy metal bacteria, Si-NPs, or MLE had significantly decreased MDA and EL contents. Moreover, the stability of cell membranes owing to H_2_O_2_-mediated membrane peroxidation (Ahanger et al., 2018), lipoxygenase activity (Heidari and Tafazoli, 2005), and polyunsaturated fatty acids (Alqarawi et al., 2014) is decreased by soil contaminated with heavy metals.

#### 3.2.4 Antioxidative defense system: Enzymatic and non-enzymatic components

**Figure 1:**
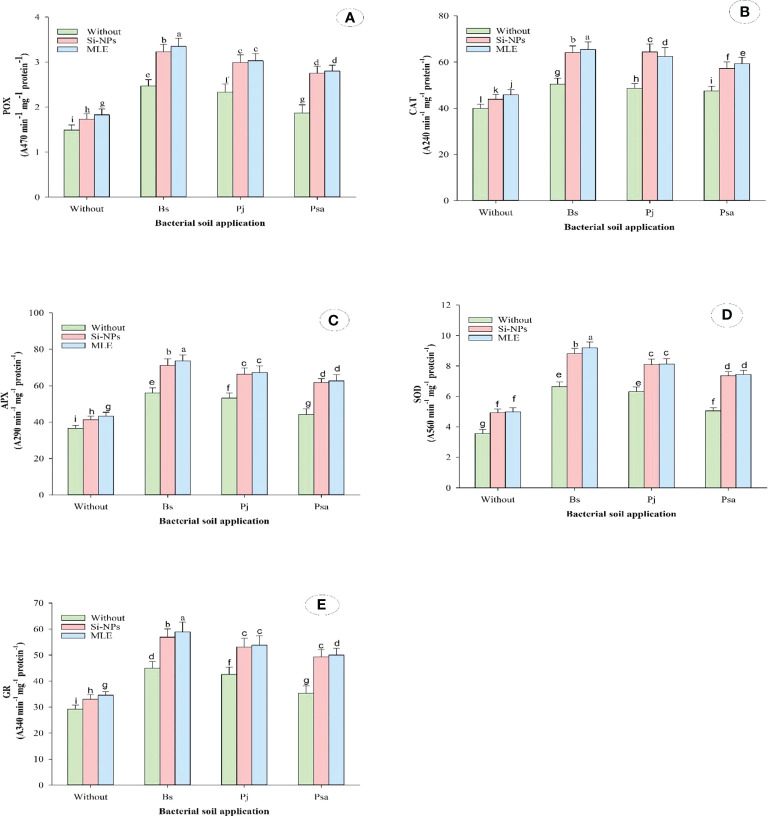
Changes in antioxidant enzymes [i.e., **(A)** peroxidase (POX), **(B)** catalase; CAT, **(C)** ascorbate peroxidase (APX), **(D)** superoxide dismutase (SOD), and **(E)** glutathione reductase (GR)] of heavy metal–stressed spinach plant in response to bioremediation of heavy metals bacterial applied as soil inoculation and/or foliar spray with MLE or Si-NPs. .

Compared with the control, adding bacteria capable of removing heavy metals from soil and applying foliar spray with MLE or Si-NPs significantly increased free proline, soluble sugars, α-TOC, GSH, AsA, and glycine betaine in spinach plants compared with untreated plants. In addition, the activities of CAT, POX, APX, SOD, and GR were also improved (**Table 6**; **Figure 1**). Furthermore, integrative applications have a superior impact on individuals. Among all the integrative treatments, the one with *B. subtilis* used in soil integrated with MLE as the foliar spray was the best, causing an increase of free proline by 49.6%, soluble sugars by 53.7%, α-TOC by 102%, GSH by 68.3%, AsA by 74.5%, glycine betaine by 56.9%, POX by 124%, CAT by 64.1%, SOD by 158%, APX by 101%, and GR by 101%.

**Table 6:** Changes in free proline, soluble sugars, α-tocopherol (α-TOC), ascorbate (AsA), and glutathione (GSH), and glycine betaine (GB) of heavy metal–stressed spinach plant in response to bioremediation of heavy metals bacterial applied as soil inoculation and/or foliar spray with MLE or Si-NPs.

		Free proline(µmol g^−1^ DW)	Soluble sugars(mg g^−1^ DW)	*α*-TOC(µmol g^−1^ DW)	AsA(µmol g^−1^ FW)	GSH(µmol g^−1^ FW)	Glycine betaine(µg g^−1^ DW)
**Effect of bacterial soil application**							
Without		27.3 ± 1.2^d^	20.2 ± 1.3^d^	2.01 ± 0.11^d^	1.17 ± 0.06^d^	1.25 ± 0.08^d^	41.4 ± 2.3^d^
Bs		35.5 ± 2.2^a^	26.9 ± 2.2^a^	3.35 ± 0.13^a^	1.66 ± 0.07^a^	1.79 ± 0.06^a^	55.3 ± 3.1^a^
Pj		34.2 ± 2.6^b^	25.6 ± 2.3^b^	3.11 ± 0.16^b^	1.59 ± 0.05^b^	1.70 ± 0.07^b^	52.5 ± 2.5^b^
Psa		32.7 ± 2.8^c^	24.4 ± 1.3^c^	2.81 ± 0.18^c^	1.51 ± 0.07^c^	1.55 ± 0.09^c^	48.8 ± 2.9^c^
**Effect of foliar spray**							
Without		29.9 ± 1.5^c^	22.0 ± 1.5^c^	2.37 ± 0.12^c^	1.32 ± 0.07^c^	1.35 ± 0.05^c^	44.2 ± 2.1^c^
Si-NPs		33.4 ± 1.6^b^	25.1 ± 1.6^b^	3.00 ± 0.18^b^	1.55 ± 0.06^b^	1.67 ± 0.03^b^	51.5 ± 2.9^b^
MLE		34.0 ± 1.8^a^	25.7 ± 1.4^a^	3.08 ± 0.17^a^	1.59 ± 0.09^a^	1.70 ± 0.08^a^	52.9 ± 2.4^a^
**Effect of interaction**							
**Soil application**	**Foliar spray**						
Without	Without	25.0 ± 1.1^g^	18.6 ± 1.1^i^	1.82 ± 0.09^i^	1.02 ± 0.07^j^	1.17 ± 0.06^k^	38.1 ± 1.9^j^
	Si-NPs	28.1 ± 1.4^f^	20.7 ± 1.2^h^	2.06 ± 0.11^h^	1.23 ± 0.04^i^	1.29 ± 0.07^j^	42.6 ± 1.6^i^
	MLE	28.7 ± 1.3^f^	21.5 ± 1.1^g^	2.16 ± 0.13^g^	1.28 ± 0.03^h^	1.31 ± 0.06^j^	43.6 ± 2.3^hi^
Bs	Without	32.3 ± 1.9^d^	23.8 ± 1.3^e^	2.80 ± 0.15^e^	1.48 ± 0.07^e^	1.47 ± 0.05^g^	48.1 ± 2.8^f^
	Si-NPs	37.0 ± 1.5^a^	28.4 ± 1.6^a^	3.56 ± 0.18^b^	1.73 ± 0.08^b^	1.94 ± 0.03^b^	58.1 ± 2.9^b^
	MLE	37.4 ± 2.1^a^	28.6 ± 1.5^a^	3.68 ± 0.14^a^	1.78 ± 0.06^a^	1.97 ± 0.08^a^	59.8 ± 2.7^a^
Pj	Without	31.6 ± 1.9^d^	23.1 ± 1.3^f^	2.66 ± 0.12^f^	1.42 ± 0.09^f^	1.42 ± 0.03^h^	45.9 ± 2.5^g^
	Si-NPs	35.2 ± 2.4^b^	26.4 ± 1.6^c^	3.32 ± 0.11^c^	1.67 ± 0.06^c^	1.82 ± 0.07^d^	55.2 ± 2.3^c^
	MLE	35.8 ± 2.5^b^	27.3 ± 1.7^b^	3.36 ± 0.16^c^	1.69 ± 0.04^c^	1.86 ± 0.06^c^	56.3 ± 2.6^c^
Psa	Without	30.7 ± 2.3^e^	22.6 ± 1.3^f^	2.20 ± 0.13^g^	1.35 ± 0.08^g^	1.34 ± 0.09^i^	44.5 ± 2.8^h^
	Si-NPs	33.5 ± 2.1^c^	25.1 ± 1.5^d^	3.08 ± 0.17^d^	1.58 ± 0.09^d^	1.64 ± 0.07^f^	50.1 ± 2.1^e^
	MLE	33.9 ± 2.6^c^	25.6 ± 1.7^d^	3.13 ± 0.13^d^	1.60 ± 0.07^d^	1.68 ± 0.08^e^	51.8 ± 2.9^d^

Data are means (n = 9) ± SE. The same letters in each column indicate no significant differences according to the LSD test (p ≤ 0.05). Without, treated with tap water; Bs, Bacillus subtilis ssp.; Pj, Paenibacillus jamilae; Psa, Pseudomonas aeruginosa; Si-NPs, silicon dioxide nanoparticles; MLE, moringa leaf extract.

Proline is also altered in plants subjected to salinity, as it contributes to osmotic adjustment. Under saline conditions, plants accumulate proline in response to salt stress (Zhu, 2001). The increase in proline accumulation in plants grown under salinity conditions may be due to acclimation to recompense for the energy for growth and survival, helping the plants in tolerating stress (Chandrashekar and Sandhyarani, 1996). Accumulating cellular proline under normal conditions has been found in about 5% of the amino acid pool, whereas accumulating cellular proline under stress conditions has been found in up to 20%–80%. This may increase synthesis and decrease degradation in many plant species (Kishor et al., 2005). Soluble sugar and proline are the primary solutes involved in osmotic adjustment in plants subjected to salinity stress (Marschner, 1995).

The increase in the antioxidant α-TOC content forms a considerable part of defense systems to adapt with the oxidative stress (Valero et al., 2016). The production of AsA and GSH augmented the activities of reactive oxygen species scavenging and metal ion chelating, forming a crucial part of abiotic stress responses in plant cells. The endogenous production of antioxidants increased the deleterious effects of Cd and/or Pb on metabolic processes and thus plant growth (Desoky et al., 2019). The antioxidants improve the complex antioxidant defense systems of plants such as cellular defense strategies against oxidative stress of heavy metals that would relieve and fix the damage from ROS overproduction (Semida et al., 2016). Ascorbate is one of the important plant antioxidants in the defense system. The ascorbate reacts with numerous reactive oxygen species such as liquid hydroperoxidases, $O_{2}^{-}$ , H_2_O_2_, and OH^−^. In addition, it is involved in numerous types of plant biological reactions that control the oxidative stresses (Conklin, 2001). The Halliwell–Asada pathway reveals that the APX uses AsA to oxidize MDA and raise DHA in chloroplasts. Afterward, the decline in both MDA and DHA will occur to rebuild the pool of ascorbate. This type of scavenging may occur near PSI to mitigate the escaping risk of ROS and also the reacting of ROS with each other (Foyer and Noctor, 2005). The biosynthesis of AsA from a hexose phosphate and its effect on the protection of photo-oxidative stress indicate that there might be links between ascorbate pool size and photosynthesis (Reddy et al., 2004). Subsequently, AsA participated to increasing the tolerance of heavy metal cucumber cultivation by increasing photosynthetic efficiency, growth, and enzymatic and non-enzymatic antioxidant activities, reducing root and leaf heavy metal contents.

Proline is considered one of the major adaptive mechanisms of the induced stress by heavy metal (Zengin and Munzuroglu, 2005). Proline mitigates the heavy metal toxicity by eliminating toxins from ROS, amplifying the contents of ASA and GSH, and promoting the activities of CAT, SOD, APX, POX, and GR (Hayat et al., 2012). In addition, proline reacts with Cd^+2^ ions in plant organs, forms a non-toxic compound of Cd–proline, and prevents membrane damage and electrolyte leakage by Cd (Semida et al., 2016).

Heavy metal stress (Cd and/or Pb) in plants results in the overproduction of ROS, which is regulated by antioxidant defense systems that include non-enzymatic antioxidants including GSH, enzymatic antioxidants (SOD, APX, CAT, POX, and GR), carotenoids, α-TOC, and proline (Semida et al., 2018). Accordingly, spinach was affordable to promote enzymatic antioxidant activities under the stress of these heavy metals. However, enzymatic and non-enzymatic compounds presented in this study reduced in the spinach leaves when applying bacteria even with the stress of Cd and/or Pb. Therefore, this study presents the first evidence about the role of *Bacillus subtilis* ssp. (MA3), *Pseudomonas aeruginosa* (SN36), and *Paenibacillus jamilae* (LA22) in producing these antioxidants (enzymatic and non-enzymatic) under Cd and/or Pb stresses.

The application of MLE increased the soluble sugar and proline of spinach plants under heavy metal stress, and this may be attributed to the fact that moringa is a good source of amino acids, soluble sugar, and antioxidants (Rady et al., 2013). In addition, the application of MLE with soil contaminated with heavy metal significantly increased the antioxidant enzymes in spinach plants (Rady et al., 2013). Moringa is a rich source of zeatin and improves chlorophyll and antioxidant enzymes (Foidl et al., 2001; Rady et al., 2013). This study recommended MLE for notable improvements in the defense capacity against oxidative damage provoked by the heavy metal in plants through increasing the activity of the antioxidant system.

#### 3.2.5 Content of heavy metal in leaf of spinach plant

**Figure 2:**
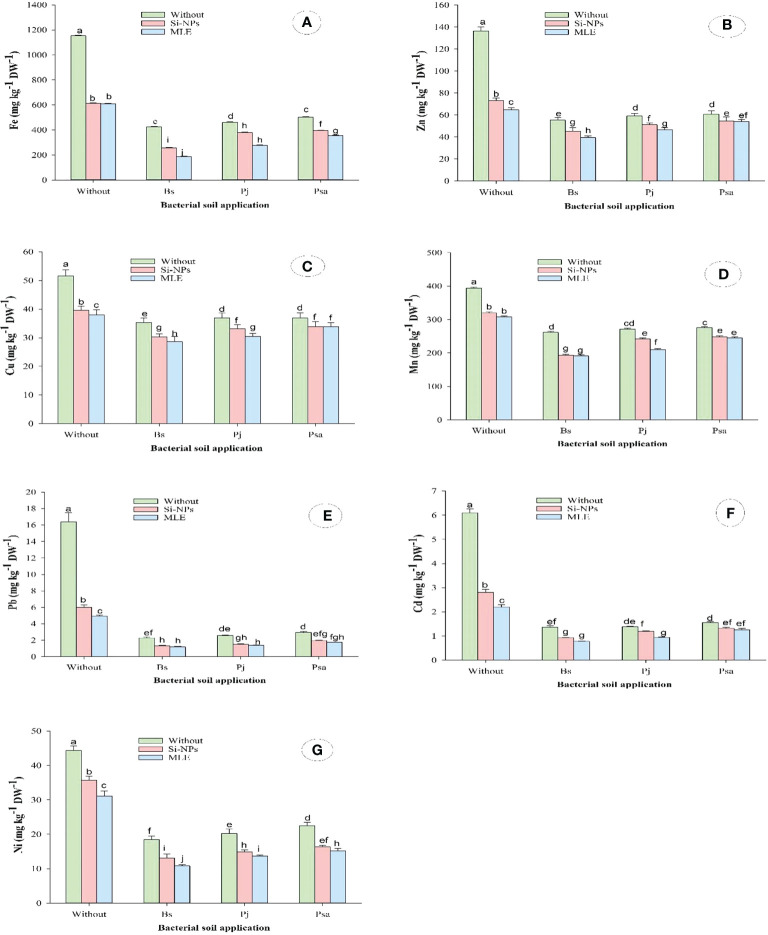
Changes in **(A)** iron (Fe), **(B)** zinc (Zn), **(C)** copper (Cu), **(D)** manganese (Mn), **(E)** lead (Pb), **(F)** cadmium (Cd), and **(G)** nickel (Ni) of heavy metal–stressed spinach plant in response to bioremediation of heavy metals bacterial applied as soil inoculation and/or foliar spray with MLE or Si-NPs. .

This study showed similar trends concerning the contents of the heavy metals zinc (Zn), iron (Fe), copper (Cu), manganese (Mn), cadmium (Cd), lead (Pb), and nickel (Ni) in spinach leaves grown on heavy metal–contaminated soil. Both single and integrative treatments significantly declined the contents of Fe, Zn, Cu, Mn, Pb, Cd, and Ni as compared with control, with superior effects of combined application than single ones. The combined application of *Bacillus subtilis* as soil amendment with MLE as foliar spray recorded the highest reductions in Fe by 83.7%, Zn by 71.1%, Cu by 44.4%, Mn by 51.4%, Pb by 92.8%, Cd by 87.2%, and Ni by 75.6%, as compared with control (**Figure 2**)

The application of bacteria for the remediation of heavy metals promotes plant cells to alter the accumulation of heavy metals, which resulted in the healthy growth of seedlings with this stress (Desoky et al., 2019). The heavy metals as non-essential elements have no specific transport channel in plants and are transported *via* transporters (Clemens et al., 1998). Therefore, heavy metals accumulated intensively in spinach roots than in leaves. Microbial metabolism can output secretions, such as organic acids of low molecular weight, which promote the solubility of heavy metals in soils (Jin et al., 2018).

The plant extracts such as MLE can be effective tools for the phytoremediation of heavy metals, as their foliar application notably reduced the accumulation of Cd, Cu, Pb, and Ni in spinach plants. The mechanism that these extracts decreased the accumulation of heavy metals in spinach plants is due to amplifying the efficiency of the antioxidant defense systems, even enzymatic or non-enzymatic, which provoke heavy metals’ entry into the plant organs (Kováčik and Dresler, 2018). Mycorrhizal symbiosis along with morphological structures such as cell walls, biologically active tissues, and thick cuticles can act as barriers to the accumulation of heavy metals and can be supported by applied extracts (Harada et al., 2010). On the other hand, the used Si-NPs in this study have a key role in decreasing the accumulation of heavy metals. In this respect, Alzahrani et al. (2018) illustrated that the addition of Si notably mitigated the Cd accumulation in wheat roots and shoots. The Si uptake mediated Cd absorption and translocations from roots to plant shoots, which promotes the plant tolerance to Cd toxicity (Wang et al., 2016).

Finally, the present study indicated that the application of remediation by heavy metal bacteria and/or foliar spray with MLE or Si-NPs succeeded to be an efficient strategy to alleviate the injuries in spinach plants that are grown under heavy metal stress due to the strong decrease in heavy metal uptake and/or in their translocation to the upper plant parts.

## 4 Conclusion

Stress tolerance in spinach plants grown on soil contaminated with heavy metals was effectively enhanced by the addition of heavy metal–resistant bacteria, i.e., *Bacillus subtilis* (SA6), *Paenibacillus* (AT26), and *Pseudomonas aeruginosa* (MM40), and/or foliar spray with MLE or Si-NPs, which play a key role in plant growth due to its beneficial influences on mineral nutrition and mechanical strength and, therefore, plant resistance to abiotic stresses. The leverage of heavy metal–resistant bacteria in combination with MLE or Si-NPs in alleviating the stress in plants reflecting better growth and yield is found to be due to the enhanced antioxidant defense systems, non-enzymatic and enzymatic antioxidants (i.e., free proline, TSS, carotenoids, CAT, POD, SOD, APX, and GR), to decline the ROS damages. The role of heavy metal–resistant bacteria and/or foliar spray with MLE or Si-NPs on supporting the antioxidative defense systems in plants under stress is also reported in our study as a “stay-green effect” due to their usage of an eco-friendly, rapid, and low-cost method.

## Data availability statement

The raw data supporting the conclusions of this article will be made available by the authors, without undue reservation.

## Author contributions

Conceived and designed the experiments: AE, E-SA, AI, A-RM, and E-SD. Performed the experiments: AE, A-RM, and E-SD. Contributed reagents/materials/analysis tools: AE, A-RM, and E-SD. Wrote the paper: AE and A-RM. Revised the paper: AE, E-SA, AI, A-RM, and E-SD. All authors contributed to the article and approved the submitted version.

## Conflict of interest

The authors declare that they have no known competing financial interests or personal relationships that could have appeared to influence the work reported in this paper.

## Publisher’s note

All claims expressed in this article are solely those of the authors and do not necessarily represent those of their affiliated organizations, or those of the publisher, the editors and the reviewers. Any product that may be evaluated in this article, or claim that may be made by its manufacturer, is not guaranteed or endorsed by the publisher.
