# Supplementary material for: Integrative application of heavy metal–resistant bacteria, moringa extracts, and nano-silicon improves spinach yield and declines its contaminant contents on a heavy metal–contaminated soil
Source: Front Plant Sci. 2022 Nov 15;13:1019014. doi: 10.3389/fpls.2022.1019014 (PMC9705991; doi:10.3389/fpls.2022.1019014)
Supplement: Supplementary file 1 [file DataSheet_1.docx]

**supplementary data**

**Table S1:** Meteorological data from the spinach cultivation in 2021

| Month | Season 2021 | | | | | |
| --- | --- | --- | --- | --- | --- | --- |
|  | Min Temp | Max Temp | Humidity | Wind | Sun | Rad |
|  | C ^o^ | C ^o^ | % | Kg/day | Hours | MJ/m^2^/day |
| October | 13.9 | 18.6 | 39.6 | 328 | 9.6 | 18.5 |
| November | 11.8 | 17.7 | 35.8 | 285 | 8.4 | 14.4 |
| December | 10.3 | 15.4 | 38.5 | 320 | 7.1 | 11.9 |

**Table S2.** Classification results rate by Bruker Daltonik MALDI Biotyper.

| **Isolate number** | **Isolate**  **code** | **analyte Name** | **Organism**  **(Best matches)** | **Value** |
| --- | --- | --- | --- | --- |
| 1 | SA6 | [H1](file:///C:\Users\admin\AppData\Roaming\Bruker%20Daltonik\MALDIBiotyperAutomationControl\HtmpResults\Bruker%20Project.html#ID0EIAA) (++) (A) | [*Bacillus subtilis* ssp. *spizizenii* DSM 15029T DSM](file:///C:\Users\admin\AppData\Roaming\Bruker%20Daltonik\MALDIBiotyperAutomationControl\HtmpResults\170430_Dr%20Mohamed.html#ID0ETCA) | 2.236 |
| 2 | AT26 | [H](file:///C:\Users\admin\AppData\Roaming\Bruker%20Daltonik\MALDIBiotyperAutomationControl\HtmpResults\Bruker%20Project.html#ID0EHAA)9 (+++) (B) | *Paenibacillus jamilae* DSM 13815T DSM | 2.554 |
| 3 | MM40 | H20 (+++) (A) | *Pseudomonas aeruginosa* A07_08_Pudu FLR | 2.631 |
